# Sociodemographic differences in Covid-19 vaccine uptake in Denmark: a nationwide register-based cohort study

**DOI:** 10.1186/s12889-023-15301-x

**Published:** 2023-02-24

**Authors:** Mie Agermose Gram, Ida Rask Moustsen-Helms, Palle Valentiner-Branth, Hanne-Dorthe Emborg

**Affiliations:** Department of Infectious Disease Epidemiology and Prevention, Statens Serum Institut, 5 Artillerivej, 2300 Copenhagen S, Denmark

**Keywords:** Covid-19 vaccination, Vaccination coverage, Sociodemographic factors, Social determinants of health, Immigrants

## Abstract

**Background:**

Covid-19 vaccination is the main strategy to reduce SARS-CoV-2 transmission, mortality and morbidity. This study aimed to examine sociodemographic differences in Covid-19 vaccine uptake among all individuals invited for Covid-19 vaccination in Denmark.

**Methods:**

This study was designed as a nationwide register-based cohort study. The study population included all Danish residents aged 12 years or above in Denmark between December 27, 2020 and October 20, 2021. Individuals who died during the study period before receiving an invitation for Covid-19 vaccination were excluded. Associations between sociodemographic factors and Covid-19 vaccination uptake were analyzed using logistic regression models adjusting for age, sex, immigration status, educational level, disposable income and history of SARS-CoV-2 infection.

**Results:**

The study population included 5,164,558 individuals. The overall vaccination coverage was 87.1% by October 20, 2021. In the full adjusted logistic regression models, the highest ORs for non-vaccination were observed among individuals aged 12–24 years (OR: 8.99 (95% CI: 8.76–9.23)), descendants of non-western immigrants (OR: 5.26 (95% CI: 5.18–5.33)), individuals who never had a PCR-test performed (OR: 2.93 (95% CI: 2.90–2.96)), individuals with primary school as highest completed educational level (OR: 2.87 (95% CI: 2.83–2.91)) and individuals with disposable income < 33,605 EUR (OR: 3.72 (95% CI: 3.52–3.93)).

**Conclusion:**

Overall, the Covid-19 vaccine uptake was high in Denmark. However, large sociodemographic differences in the vaccine uptake exist. The youngest age groups had the lowest vaccination coverage. Furthermore, the impact of the sociodemographic factors was more pronounced in the youngest age groups. The identified determinants may be used to design policies to help maximize the vaccination coverage.

**Supplementary Information:**

The online version contains supplementary material available at 10.1186/s12889-023-15301-x.

## Background

A key challenge is to ensure sufficient vaccine uptake in the population to reduce SARS-CoV-2 transmission, mortality and morbidity from Covid-19 [1]. A national Covid-19 vaccination program was introduced in Denmark on 27 December 2020. The Danish Health Authority prioritized nursing home residents, individuals aged 85 years or above, vulnerable and frontline personnel first for vaccination followed by age groups younger than 85 years in decreasing order. By October 20, 2021, 87.1% of the Danish population invited for vaccination (individuals aged 12 years or above) had received at least the first Covid-19 vaccine dose. Understanding which sociodemographic, factors are associated with low vaccination coverage has major implications for designing policies that help maximize the vaccination coverage. Previous studies in this field of research have investigated attitudes and potential acceptance toward Covid-19 vaccination [2–4]. Showing clear sociodemographic differences in intention to accept a vaccine for Covid-19 with the intention being higher in those with higher income levels [3, 4] and higher education levels [2–4]. A Scottish study showed that white ethnicity was positively associated with vaccine acceptance compared with Black, Asian, and minority ethnic groups [4]. However, a study from the US showed that especially Asian and American Indian/Alaska Native racial groups were positively associated with Covid-19 vaccine acceptance compared to Black/African Americans [2]. Sociodemographic differences in actual Covid-19 vaccination uptake have only been examined in older adults [5–7]. These studies demonstrate that the greatest disparities in Covid-19 vaccine uptake were observed with younger age [7], male sex [5], lower income [5–7] and living alone [6, 7] certain religious groups [6] and between ethnic groups [5, 6]. It is important to examine associations between sociodemographic factors and Covid-19 vaccine uptake for all ages because other associations may exist in younger age groups. The aim of this study was to examine sociodemographic differences in Covid-19 vaccine uptake among all individuals invited for Covid-19 vaccination in Denmark.

## Methods

### Study design and population

The study was designed as a nationwide register-based cohort study. The study population included all residents invited for Covid-19 vaccination (individuals aged 12 years or above) in Denmark between December 27, 2020 and October 20, 2021. Individuals who died during the study period before receiving an invitation for Covid-19 vaccination were excluded.

### Data sources

Individual-based data from nationwide Danish registries were linked using the unique personal identification number collected from the Danish Civil Registration System (CRS) [8]. Information on sex, date of birth, immigration status, geographical region of residence, marital status and number of individuals in the household was obtained from CRS [8]. All administered Covid-19 vaccines are registered in the Danish Vaccination Registry (DVR) on an individual level, identified by the CRS number [9]. Information on educational level and disposable income were obtained from Statistics Denmark [10]. Information about previous SARS-CoV-2 infections (sample date and test results) was obtained from the Danish Microbiology Database (MiBa) [11]. MiBa is a national database containing real-time information on all microbiological laboratory test results from all clinical microbiology departments and private test centers. Information on chronic diseases within 5 years prior to study entry was obtained from the Danish National Patient Registry (DNPR) [12]. For each hospital contact, one primary and one or more optional secondary diagnoses are provided and coded according to the International Classification of Diseases, 10th revision (ICD-10) [12]. The ICD-10 codes used to define the included chronic diseases are shown in Supplementary (Table S2). The date of death for exclusion before invitation to Covid-19 vaccination was obtained from CRS [8]. All data were uploaded to Statistics Denmark for data management and analysis.

### Outcome

The outcome in this study was having received at least one dose of a Covid-19 vaccine (BNT162b2 mRNA, mRNA-1273, ChAdOx1 or Ad26.COV2.S) between December 27, 2020 and 20 October, 2021.

### Sociodemographic variables

The sociodemographic characteristics included in this study as predictors for non-vaccination were sex, age, immigration status, chronic diseases, infection status, educational level, disposable income, geographical region, marital status and living alone/not alone. Sex was defined as female or male. The age groups were divided into 12-15 years, 16–24 years, 25–34 years, 35–44 years, 45–54 years, 55–64 years, 65–74 years, 75–84 years and 85 years and above. Covid-19 vaccination was approved later for individuals aged 12–15 years (May 2021) [13]. Although the vaccine was approved for this age group while the vaccine roll-out was ongoing, the 12–15 years old were the last in Denmark to be invited for Covid-19 vaccination (July 2021). Therefore, we examined this age group separately. Immigration status was defined as Danish, immigrants of western descent, descendants of western immigrants, immigrants of non-western descent, descendants of non-western immigrants (see Supplementary, Table S1). Chronic diseases were defined as having at least one chronic disease or no chronic disease (see supplementary, Table S2). History of SARS-CoV-2 infection was defined as no history of SARS-CoV-2 infection before vaccination, history of SARS-CoV-2 infection before vaccination and never PCR-tested. The infections were diagnosed by PCR tests. The highest completed educational level was defined as master or Ph.D., bachelor, secondary school, vocational school and primary school. Disposable income was defined as 1,000,000 DDK or above (> 134,416 EUR), 700,000-999,999 DDK (94,091–134,416 EUR), 450,000-699,999 DDK (60,487 − 94,090 EUR), 250,000-449,999 DDK (33,605 − 60,486 EUR) and < 250,000 DDK (< 33,605 EUR). The income was converted to EUR to give a better perception. The exchange rate for 1 EUR was 7,44 DDK on February 2023 according to the central bank of Denmark. The majority of the population is represented in the lowest income group. However, we did not have information about the exact disposable income. Therefore, we were not able to create another division. The geographical region of residence was defined as Capital Region of Denmark, Region Zealand, Northern Denmark Region, Central Denmark Region and Region of Southern Denmark. Finally, marital status was defined as with a partner, divorced or widow/widower and unmarried) and living alone was defined as living alone and not living alone.

### Statistical analysis

**Fig. 1:**
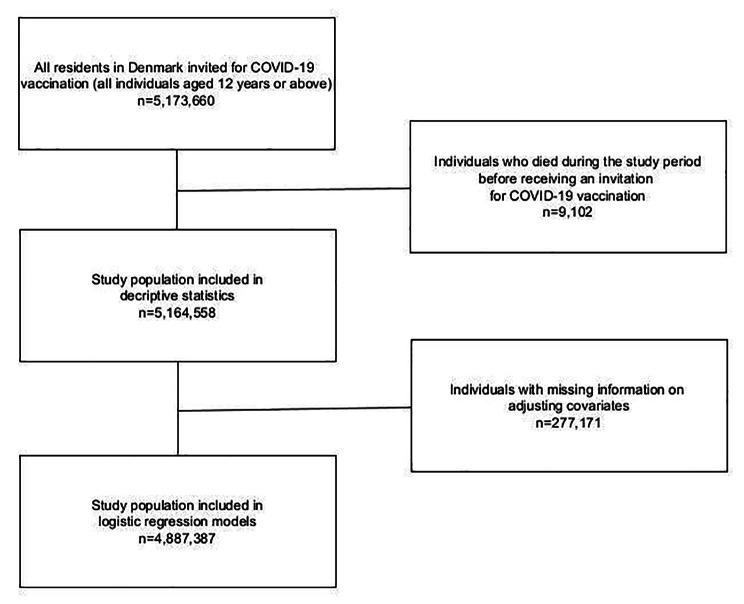
Flow chart of the study population

For each level of the sociodemographic variables numbers and proportions of vaccinated were calculated. In addition, stratified analyses presenting proportions vaccinated across sociodemographic variables were prepared to explore the combined effect of the different variables. Four logistic regression models were used to estimate odds ratio (OR) with 95% confidence intervals (95% CI) to explore the associations between each sociodemographic factor and non-vaccination. The first model was unadjusted. The second model was adjusting for age groups and sex. The third model was adjusting for age, sex, immigration status, educational level and disposable income. Finally, the fourth model was adjusting for age, sex, immigration status, educational level, disposable income and history of SARS-CoV-2 infection. Individuals were excluded from the logistic regression models if any information on the adjusting sociodemographic factors was missing (Fig. 1). This exclusion was done to guarantee that the same number of individuals were included in all four models to ensure that changes in the OR estimates were explained by adjustment and not differences in the included individuals. Coefficients and standard error (SE) from the logistic regression models were reported for educational level and disposable income to examine any collinearity (See supplementary, Table S3).

All analyses were performed using R statistical software version 2022.02.1.

## Results

The study population for the descriptive statistics included 5,164,558 residents in Denmark invited for Covid-19 vaccination (12 years or above) and the study population for the logistic regression models included 4.887.387 individuals without missing values on the sociodemographic covariates used for adjustment (Fig. 1).

**Fig. 2:**
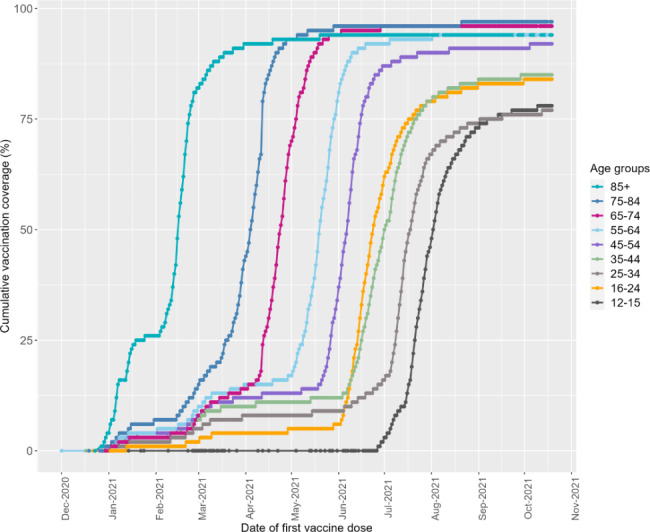
Daily cumulative proportion vaccinated by age groups

The oldest age groups were vaccinated in the early stage of the vaccination program from end of December 2020 until mid-April 2021 and reached a higher vaccination coverage than the younger age groups. All age groups have been invited for vaccination by July 2021 (Fig. 2).

### Descriptive statistics

**Fig. 3:**
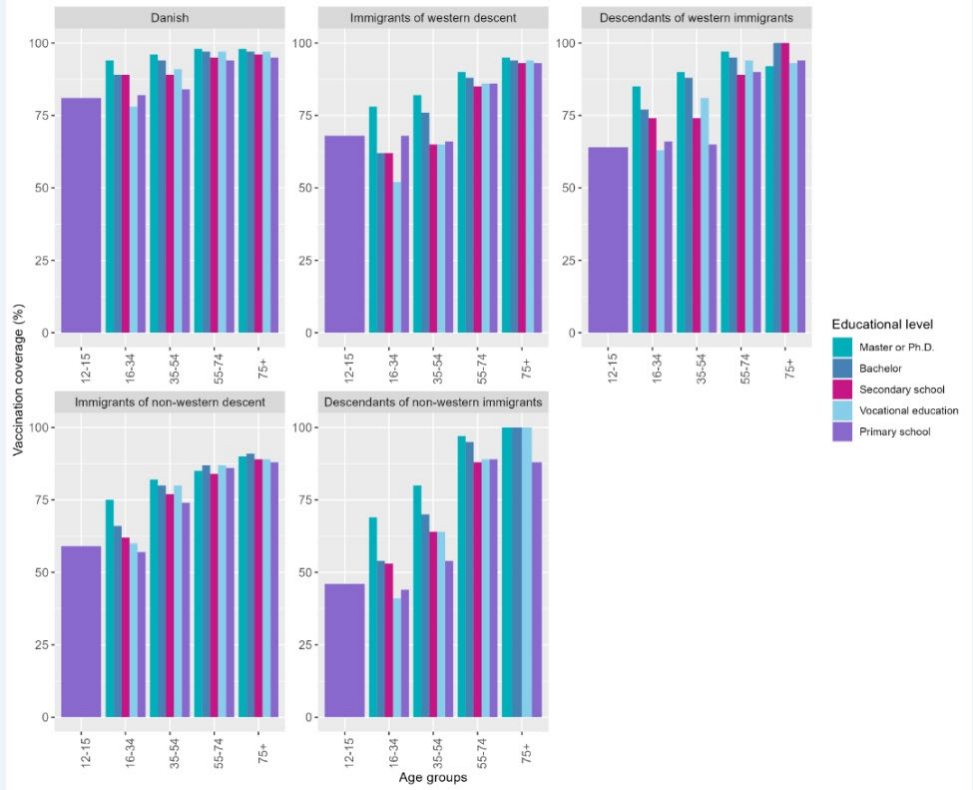
Proportion vaccinated stratified by immigration status, age groups and educational level

**Fig. 4:**
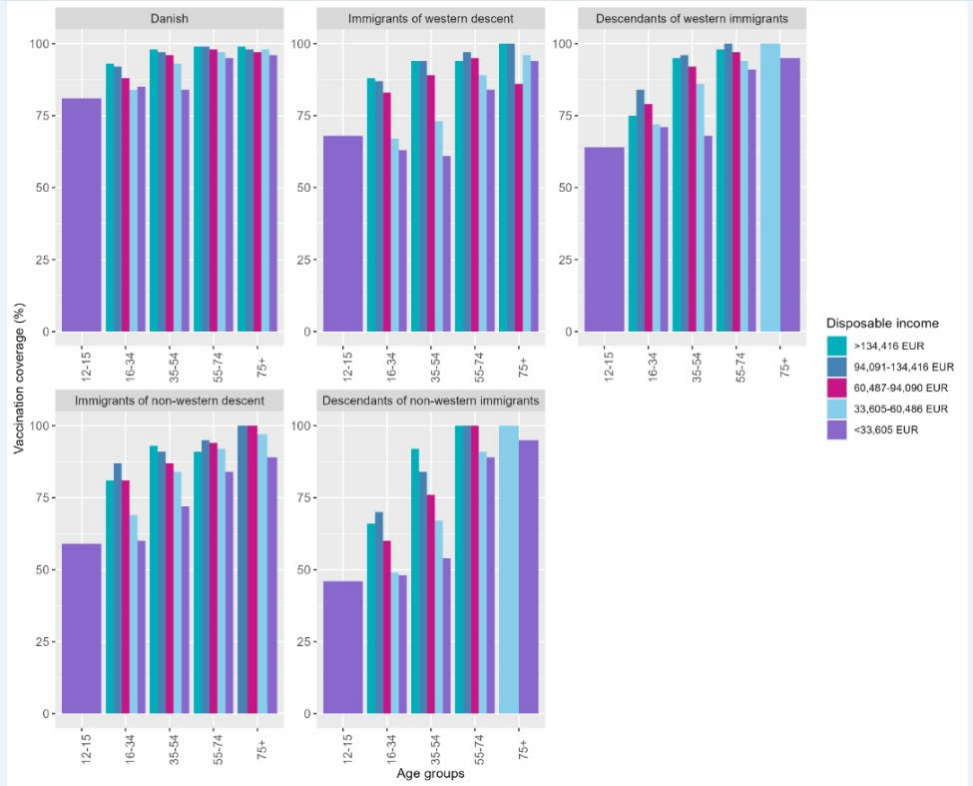
Proportion vaccinated stratified by immigration status, age groups and income

**Fig. 5:**
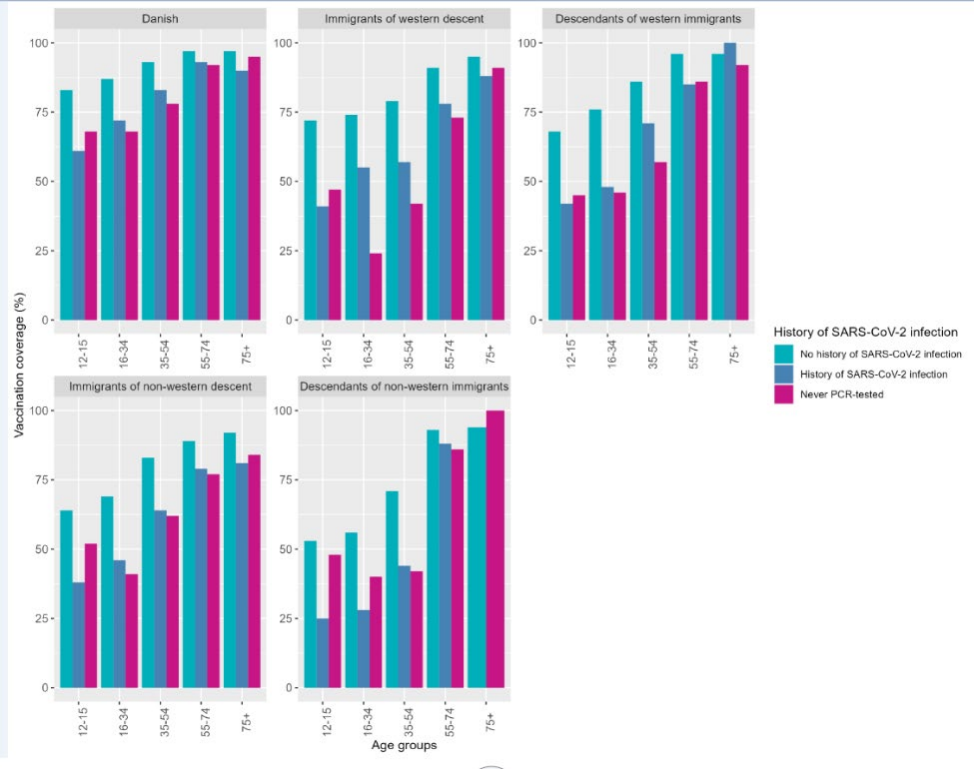
Proportion vaccinated stratified by immigration status, age groups and history of SARS-CoV-2 infection

The mean age in the study population was 47 (SD: 20.7) years and 50.5% were women. By October 20, 2021, 87.1% of the study population had received at least the first dose of a Covid-19 vaccine. The vaccination coverage was similar among males and females. The vaccination coverage was lower among younger age groups: 12–15 years (70.0% vaccinated), 16–24 years (82.0% vaccinated), 25–34 years (75.7% vaccinated) and 35–44 years (84.1% vaccinated) (Table 1). Furthermore, individuals with other immigration status than Danish had lower vaccination coverage: immigrants of western descent (68.5% vaccinated), descendants of western immigrants (73.9% vaccinated), immigrants of non-western descent (73.9% vaccinated) and descendants of non-western immigrants (49.2% vaccinated). Lower vaccination coverage was also observed among individuals without chronic diseases (86.0% vaccinated), individuals with upper secondary school (85.0% vaccinated) or primary school (83.9% vaccinated) as highest completed educational level, disposable income < 33,605 EUR (85.0% vaccinated) and among individuals with history of SARS-CoV-2 infection before vaccination (79.5% vaccinated) and individuals who never had been PCR-tested (69.6% vaccinated). Vaccine uptake was lower among unmarried individuals (79.3% vaccinated). The vaccine uptake was 88.9% and 87.0% among individuals living alone and not living alone, respectively. The vaccination coverage was ranging between 85.5% and 88.7% across all five geographical regions (Table 1). Larger differences in vaccine uptake exist within sociodemographic factors across age groups and immigration status. Especially immigrants and descendants of immigrants aged 12–15 years, 16–34 years or 35–54 years had a lower vaccine uptake (Figs. 3, 4 and 5). Descendants of non-western immigrants aged 16–34 years with primary school as the highest educational level had a vaccine uptake of 41% whereas the vaccine uptake was 69% among descendants of non-western immigrants with master or Ph.D levels. However, the highest vaccine uptake within all educational levels in descendants of non-western immigrants is lower than the lowest vaccine uptake in Danish individuals regardless of educational level (Fig. 3). Among immigrants of western descent aged 16–34 years only 24% were vaccinated if they had history of SARS-CoV-2 infection whereas 74% were vaccinated if they had no history of SARS-CoV-2 infection.

**Table 1:** Characteristics of the study population

	Number of individuals in the study population	Percentage of study population	Number of vaccinated individuals	Percentage vaccinated individuals
**Total**	5,164,558	100.0	4,497,939	87.1
**Sex**				
Female	2,606,628	50.5	2,289,752	87.8
Male	2,557,930	49.5	2,208,185	86.3
**Age groups**				
85 years or above	130,221	2.5	122,573	94.1
75–84 years	425,780	8.2	411,028	96.5
65–74 years	640,675	12.4	616,391	96.2
55–64 years	759,604	14.7	713,715	94.0
45–54 years	797,148	15.4	726,376	91.1
35–44 years	688,535	13.3	579,147	84.1
25–34 years	779,232	15.1	589,995	75.7
16–24 years	654,837	12.7	536,775	82.0
12–15 years	288,526	5.6	201,937	70.0
**Immigration status**				
Danish	4,330,606	83.9	3,927,072	90.7
Immigrants of western descent	341,830	6.6	23,4192	68.5
Descendants of western immigrants	153,00	0.3	11,311	73.9
Immigrants of non-western descent	367,200	7.1	271,363	73.9
Descendants of non-western immigrants	108,475	2.1	53,394	49.2
Unknown	1,147	0.0	605	52.7
**Chronic diseases**				
No	4,072,160	78.8	3,500,074	86.0
Yes	1,092,398	21.2	997,863	91.3
**History of SARS-CoV-2 infection**				
Never PCR-tested	281,593	5.5	195,963	69.6
No history of SARS-CoV-2 infection before vaccination	4,144,359	80.2	3,714,669	89.6
History of infection before vaccination	738,606	14.3	587,314	79.5
**Educational level**				
Master or Ph.D.	504,801	9.8	468,334	92.8
Bachelor	1,056,670	20.5	959,903	90.8
Secondary school	455,243	8.8	386,749	85.0
Vocational education	1,466,981	28.4	1,328,168	90.5
Primary school	1,427,247	27.6	1,197,790	83.9
Unknown	253,616	4.9	156,993	61.9
**Disposable income**				
> 134,416 EUR	5,6420	1.1	54,998	97.5
94,091–134,416 EUR	129,123	2.5	125,098	96.9
60,487 − 94,090 EUR	597,733	11.6	565,503	94.6
33,605 − 60,486 EUR	1,179,001	22.8	1,056,708	89.6
< 33,605 EUR	3,130,260	60.6	2,660,256	85.0
Unknown	72,021	1.4	35,374	49.1
**Geographical region**				
Capital Region of Denmark	1,630,644	31.6	1,394,295	85.5
Region Zealand	747,878	14.5	648,979	86.8
Northern Denmark Region	524,719	10.2	465,418	88.7
Central Denmark Region	1,173,827	22.7	1,039,383	88.5
Region of Southern Denmark	1,083,918	21.0	949,705	87.6
Unknown	3,572	0.1	157	4.4
**Marital status**				
With a partner	2,157,749	41.8	2,012,761	93.3
Divorced or widow/widower	849,244	16.4	781,020	92.0
Unmarried	2,146,249	41.6	1,701,469	79.3
Unknown	11,316	0.2	2,687	23.7
**Living alone**				
Yes	1,106,565	21.4	983,810	88.9
No	3,958,429	76.6	3,442,787	87.0
Unknown	99,564	1.9	71,340	71.7

### Results from logistic regression models

The unadjusted logistic regression models showed that male sex was associated with an OR of 1.14 (95% CI: 1.13–1.14) for non-vaccination compared to females. The OR estimate was slightly lower after full adjustment (model 4) (Table 2). The highest OR for non-vaccination was observed among individuals aged 12–15 years, 16–24 years, 25–34 years and 35–44 years compared to individuals aged 85 years or above. Large differences in the OR estimates for the associations between age groups and non-vaccination was observed before and after adjustment for sociodemographic covariates. In the unadjusted model, OR was 5.20 (95% CI: 5.07–5.33) for individuals aged 25–34 years and 3.04 (95% CI: 3.00-3.12) for individuals aged 35–44 years. In the full adjusted model (model 4) the OR estimates increased to 8.99 (95% CI: 8.76–9.23) for individuals aged 25–34 years and 6.09 (95% CI: 5.94–6.26) for individuals aged 35–44 years (Table 2). Immigrants of western and non-western descent and descendants of western and non-western immigrants had higher ORs of non-vaccination compared to Danish. The highest OR was observed among descendants of non-western immigrants with an OR of 10.38 (95% CI: 10.24–10.51) in the unadjusted model and 5.26 (95% CI: 5.18–5.33) in the full adjusted model (model 4) (Table 2). The OR estimates for the associations between immigrants of western and non-western descent and descendants of western immigrants and non-vaccination were similar before and after adjustments. Disposable income <33,605 EUR was associated with the highest OR of non-vaccination. The unadjusted model showed an OR of 6.29 (95% CI: 5.97–6.64) and the full adjusted model (model 4) showed an OR of 3.72 (95% CI: 3.52–3.93) compared to income above 134,416 EUR (Table 2). Smaller differences in the OR estimates between the models before and after adjustment was observed for history of SARS-CoV-2 infection and educational level. In the full adjusted model (model 4) vocational education was associated with an OR of 2.25 (95% CI: 2.22–2.28) and primary school was associated with an OR of 2.87 (95% CI: 2.83–2.91) for non-vaccination compared to master or Ph.D. level (Table 2). In the adjusted model (model 3), OR was 2.91 (95% CI: 2.88–2.93) for individuals with history of SARS-CoV-2 infection before vaccination and 2.93 (95% CI: 2.90–2.96) for individuals who had never been PCR tested compared to individuals with no history of SARS-CoV-2 infection before vaccination (Table 2). The SE were unchanged when including sociodemographic variables, which indicates that the educational level and income variable are not collinear (Table S3).

**Table 2:** Unadjusted and adjusted odds ratios (ORs) with 95% confidence interval (CI) for non-vaccination by sociodemographic factors

	Model 1^a^ OR (95% CI)	Model 2^b^ OR (95% CI)	Model 3^c^ OR (95% CI)	Model 4^d^ OR (95% CI)
**Sex**				
Female	Reference	Reference	Reference	Reference
Male	1.14 (1.13–1.14)	1.10 (1.10–1.11)	1.15 (1.14–1.15)	1.08 (1.07–1.08)
**Age groups**				
85 years or above	Reference	Reference	Reference	Reference
75–84 years	0.59 (0.57–0.60)	0.58 (0.56–0.60)	0.51 (0.59–0.63)	0.65 (0.63–0.67)
65–74 years	0.65 (0.63–0.67)	0.64 (0.63–0.66)	0.74 (0.72–0.76)	0.85 (0.83–0.88)
55–64 years	1.04 (1.01–1.07)	1.03 (1.00-1.05)	1.54 (1.50–1.58)	1.90 (1.85–1.95)
45–54 years	1.56 (1.53–1.61)	1.54 (1.51–1.58)	2.56 (2.40–2.53)	3.17 (3.09–3.26)
35–44 years	3.04 (3.00-3.12)	3.00 (2.93–3.08)	4.51 (4.39–4.62)	6.09 (5.94–6.26)
25–34 years	5.20 (5.07–5.33)	5.12 (5.00-5.25)	6.48 (6.32–6.65)	8.99 (8.76–9.23)
16–24 years	3.30 (3.22–3.38)	3.25 (3.17–3.33)	2.36 (2.30–2.42)	3.39 (3.30–3.48)
12–15 years	4.84 (4.71–4.97)	4.77 (4.64–4.90)	2.91 (2.83-3.00)	4.43 (4.30–4.56)
**Immigration status**				
Danish	Reference	Reference	Reference	Reference
Immigrants of western descent	3.83 (3.80–3.87)	3.19 (3.16–3.22)	3.50 (3.47–3.53)	3.32 (3.29–3.35)
Descendants of western immigrants	3.08 (2.95–3.21)	2.26 (2.16–2.35)	2.28 (2.18–2.38)	2.24 (2.15–2.34)
Immigrants of non-western descent	3.53 (3.50–3.56)	3.09 (3.06–3.12)	2.47 (2.45–2.49)	2.29 (2.26–2.31)
Descendants of non-western immigrants	10.38 (10.24–10.51)	6.07 (5.99–6.15)	5.79 (5.71–5.87)	5.26 (5.18–5.33)
**Chronic diseases**				
Yes	Reference	Reference	Reference	Reference
No	1.58 (1.57–1.59)	1.04 (1.03–1.04)	1.16 (1.15–1.17)	1.09 (1.08–1.10)
**History of SARS-CoV-2 infection**				
No history of SARS-CoV-2 infection before vaccination	Reference	Reference	Reference	-
Never PCR-tested	3.93 (3.89–3.96)	3.37 (3.34–3.40)	2.93 (2.90–2.96)	-
History of infection before vaccination	2.01 (1.99–2.02)	3.58 (3.55–3.61)	2.91 (2.88–2.93)	-
**Educational level**				
Master or Ph.D.	Reference	Reference	Reference	Reference
Bachelor	1.29 (1.27–1.30)	1.54 (1.52–1.56)	1.48 (1.46–1.50)	1.49 (1.47–1.51)
Secondary school	2.29 (2.26–2.33)	2.44 (2.40–2.47)	1.92 (1.89–1.95)	1.86 (1.83–1.89)
Vocational education	1.37 (1.35–1.38)	2.17 (2.14–2.20)	2.27 (2.24–2.30)	2.25 (2.22–2.28)
Primary school	2.51 (2.48–2.54)	3.82 (3.77–3.87)	3.00 (2.96–3.04)	2.87 (2.83–2.91)
**Disposable income**				
> 134,416 EUR	Reference	Reference	Reference	Reference
94,091–134,416 EUR	1.26 (1.18–1.34)	1.17 (1.10–1.24)	1.10 (1.04–1.18)	1.10 (1.03–1.17)
60,487 − 94,090 EUR	2.23 (2.11–2.36)	1.92 (1.82–2.03)	1.63 (1.54–1.72)	1.58 (1.49–1.67)
33,605 − 60,486 EUR	4.52 (4.29–4.77)	3.66 (3.47–3.86)	2.52 (2.38–2.66)	2.38 (2.25–2.51)
< 33,605 EUR	6.29 (5.97–6.64)	7.45 (7.06–7.86)	4.20 (3.97–4.43)	3.72 (3.52–3.93)
**Geographical region**				
Capital Region of Denmark	Reference	Reference	Reference	Reference
Region Zealand	0.91 (0.91–0.92)	1.08 (1.07–1.09)	1.12 (1.11–1.13)	1.11 (1.10–1.12)
Northern Denmark Region	0.75 (0.75–0.76)	0.83 (0.82–0.83)	0.88 (0.87–0.89)	0.93 (0.92–0.94)
Central Denmark Region	0.75 (0.74–0.75)	0.78 (0.78–0.79)	0.82 (0.81–0.82)	0.83 (0.83–0.84)
Region of Southern Denmark	0.83 (0.82–0.83)	0.93 (0.92–0.93)	0.93 (0.92–0.94)	0.96 (0.95–0.97)
**Marital status**				
With a partner	Reference	Reference	Reference	Reference
Divorced or widow/widower	1.26 (1.25–1.28)	1.84 (1.82–1.85)	1.66 (1.64–1.67)	1.65 (1.63–1.67)
Unmarried	3.36 (3.33–3.38)	1.92 (1.90–1.93)	1.85 (1.84–1.87)	1.75 (1.73–1.76)
**Living alone**				
No	Reference	Reference	Reference	Reference
Yes	0.91 (0.90–0.91)	1.27 (1.26–1.28)	1.25 (1.24–1.26)	1.20 (1.19–1.21)

a: Model 1: Unadjusted

b: Model 2: Adjusted for age and sex

c: Model 3: Adjusted for age, sex, immigration status, educational level and disposable income

d: Model 4: Adjusted for age, sex, immigration status, educational level, disposable income and history of SARS-CoV-2 infection

## Discussion

This study contributed with new insights into the understanding of the sociodemographic complexity in Covid-19 vaccine uptake, showing marked differences in the uptake not only between, but also within sociodemographic groups. Non-vaccination was most pronounced among individuals of younger age, immigrants or descendants, individuals with low educational level, low income, history of SARS-CoV-2 infection and never PCR-tested. However, the impact of educational level, disposable income and history of SARS-CoV-2 infection was more pronounced in the youngest age groups 12–15, 16–34 and 35–54 years of age. Previous studies have only examined sociodemographic differences in Covid-19 vaccination uptake among older adults [5–7] whereas our study contributes with evidence of sociodemographic differences in vaccine uptake among both children and adults. However, a Swedish register-based cross-sectional study of adults aged 60 years or above showed that younger age (60–74 years compared to ≥ 75 years), low income and living alone were associated with non-vaccination [7], which is similar to our findings. Their results showed that low income was associated with an OR of 2.16 (95% CI: 2.10–2.23) for non-vaccination compared to medium/high income. High-income countries and low-middle-income countries of birth were associated with ORs of 2.15 (95% CI: 2.07–2.23) and 3.86 (3.71-4.00) for non-vaccination compared to Sweden as country of birth. Living alone was associated with an OR of 1.64 (95% CI: 1.59–1.68) for non-vaccination compared to not living alone. Furthermore, the Swedish study also observed a higher proportion of non-vaccinated individuals among those with history of Covid-19. A Danish study has demonstrated that previously infected individuals also benefit from COVID-19 vaccination [14], which underlines the importance that results from these studies are taken into account when designing future policies to increase vaccination uptake in these groups. Studies from England and Wales have shown marked differences in Covid-19 vaccine uptake between ethnic groups [5, 6]. These associations are consistent with our findings. Although, the ethnic groups are not necessarily similar to immigrants and descendants in Denmark in terms of country of origin or religious affiliation. Qualitative research is needed to examine underlying explanations for non-vaccination in the identified populations with low vaccine uptake. Promoting vaccine uptake among the identified populations with low vaccine uptake should include targeted multilingual campaigns and specific outreach strategies.

The observed differences between sociodemographic factors also exist for seasonal influenza, pneumococcal and herpes zoster vaccine uptake [15, 16]. In addition to the importance of sociodemographic factors, a Danish study demonstrated the importance of health communication [17]. The results showed that vaccine acceptance increased when individuals were exposed to transparent positive communication and transparent neutral communication. Whereas transparent negative communication and vague communication decrease vaccine acceptance [17]. These are both important factors when planning future vaccination campaigns.

### Strengths and limitations

The strengths of this study consist of including all residents in Denmark invited for Covid-19 vaccination. Furthermore, data are register-based with high quality and completeness, resulting in few missing data.

We were able to include information on several sociodemographic characteristics. However, some factors were categorized into broad categories. This may have caused some of the finer differences in non-vaccination between groups to remain undetected. Furthermore, other unmeasured factors such as religion, attitude towards vaccination and trust in health authorities may also be important factors for the vaccine uptake. We identified several characteristics of non-vaccination. However, it may be difficult to identify individuals with e.g. low educational level, low disposable income or history of SARS-CoV-2 infection. Further research should identify more specific characteristics such as occupation to identify individuals with low vaccine uptake and to plan interventions or campaigns for these groups. Due to the data delivery processes, data availability only extends to October 20, 2021, and we are unable to assess if vaccination rates in certain groups indeed increased over time after this date.

In conclusion, the results demonstrated large sociodemographic differences in Covid-19 vaccination uptake, especially in the younger age groups, which have not previously been included in this field of research. Promoting vaccine uptake should include targeted multilingual campaigns and specific outreach strategies.

## Electronic supplementary material

Below is the link to the electronic supplementary material.

Supplementary Material 1

## Data Availability

The data that support the findings of this study are available from Statistics Denmark, the Danish Data Protection Agency and Danish Health and Medicines Authority but restrictions apply to the availability of these data, which were used under license for the current study, and so are not publicly available. Data are however available from the authors upon reasonable request and with permission of from Statistics Denmark, the Danish Data Protection Agency and Danish Health and Medicines Authority.

## References

[1] Strategy to Achieve Global Covid-19 Vaccination by mid-2022.

[2] Malik AA, McFadden SM, Elharake J, Omer SB (2020). Determinants of COVID-19 vaccine acceptance in the US. EClinicalMedicine.

[3] Lazarus JV, Ratzan SC, Palayew A, Gostin LO, Larson HJ, Rabin K (2021). A global survey of potential acceptance of a COVID-19 vaccine. Nat Med.

[4] Williams L, Flowers P, McLeod J, Young D, Rollins L, The Catalyst Project T. Social Patterning and Stability of Intention to Accept a COVID-19 Vaccine in Scotland: Will Those Most at Risk Accept a Vaccine? Vaccines (Basel). 2021;9(1). 10.3390/vaccines9010017 PMC7824425 33406762

[5] Perry M, Akbari A, Cottrell S, Gravenor MB, Roberts R, Lyons RA (2021). Inequalities in coverage of COVID-19 vaccination: a population register based cross-sectional study in Wales, UK. Vaccine.

[6] Nafilyan V, Dolby T, Razieh C, Gaughan CH, Morgan J, Ayoubkhani D (2021). Sociodemographic inequality in COVID-19 vaccination coverage among elderly adults in England: a national linked data study. BMJ Open.

[7] Spetz M, Lundberg L, Nwaru C, Li H, Santosa A, Leach S (2022). The social patterning of Covid-19 vaccine uptake in older adults: a register-based cross-sectional study in Sweden. Lancet Reg Health Eur.

[8] Schmidt M, Pedersen L, Sørensen HT (2014). The danish Civil Registration System as a tool in epidemiology. Eur J Epidemiol.

[9] Grove Krause T, Jakobsen S, Haarh M, Mølbak K. The Danish vaccination register.Euro Surveill. 2012;17(17). 10.2807/ese.17.17.20155-en 22551494

[10] Thygesen L (1995). The register-based system of demographic and social statistics in Denmark. Stat J UN Econ Comm Eur.

[11] Schønning K, Dessau RB, Jensen TG, Thorsen NM, Wiuff C, Nielsen L (2021). Electronic reporting of diagnostic laboratory test results from all healthcare sectors is a cornerstone of national preparedness and control of COVID-19 in Denmark. Apmis.

[12] Schmidt M, Schmidt SA, Sandegaard JL, Ehrenstein V, Pedersen L, Sørensen HT (2015). The danish National Patient Registry: a review of content, data quality, and research potential. Clin Epidemiol.

[13] First COVID-19 vaccine approved for children aged 12 to 15 in EU: European Medicines Agency; 2021 [cited 2023 January 31]. Available from: https://www.ema.europa.eu/en/news/first-covid-19-vaccine-approved-children-aged-12-15-eu.

[14] Nielsen KF, Moustsen-Helms IR, Schelde AB, Gram MA, Emborg H-D, Nielsen J et al. Vaccine effectiveness against SARS-CoV-2 reinfection during periods of Alpha (B.1.1.7), Delta (B.1.617.2) or Omicron (B.1.1.529) dominance: A Danish nationwide study. medRxiv. 2022:2022.06.01.22275858. 10.1371/journal.pmed.1004037 PMC9681105 36413551

[15] Jain A, van Hoek AJ, Boccia D, Thomas SL (2017). Lower vaccine uptake amongst older individuals living alone: a systematic review and meta-analysis of social determinants of vaccine uptake. Vaccine.

[16] La EM, Trantham L, Kurosky SK, Odom D, Aris E, Hogea C (2018). An analysis of factors associated with influenza, pneumoccocal, tdap, and herpes zoster vaccine uptake in the US adult population and corresponding inter-state variability. Hum Vaccin Immunother.

[17] Petersen MB, Bor A, Jørgensen F, Lindholt MF (2021). Transparent communication about negative features of COVID-19 vaccines decreases acceptance but increases trust. Proc Natl Acad Sci U S A.

[18] Bekendtgørelse af sundhedsloven. : The Danish Ministry of Health; [cited 2022 September 9]. Available from: https://www.retsinformation.dk/eli/lta/2019/903.

