# Supplementary material for: Sociodemographic differences in Covid-19 vaccine uptake in Denmark: a nationwide register-based cohort study
Source: BMC Public Health. 2023 Feb 24;23:391. doi: 10.1186/s12889-023-15301-x (PMC9955526; doi:10.1186/s12889-023-15301-x)
Supplement: Supplementary file 1 — Supplementary Material 1 [file 12889_2023_15301_MOESM1_ESM.docx]

## Supplementary

**Table S1. Definition of immigration status.**

|  | **Definition** |
| --- | --- |
| Danish | Individuals born in Denmark or abroad with at least one parent who is both a Danish citizen and born in Denmark. |
| Immigrants of western* descent | Individuals born abroad, where neither parent is a Danish citizen or born in Denmark. If there is no information about any of the parents and the person was born abroad, the person is also perceived as an immigrant. |
| Descendants of western* immigrants | Individuals born in Denmark, where neither parent is both a Danish citizen and born in Denmark. If there is no information about any of the parents and the person is a foreign citizen, the person is also perceived as a descendant. When one or both parents born in Denmark obtain Danish citizenship, their children will not be classified as descendants, but as persons of Danish origin. However, if Danish-born parents both maintain a foreign citizenship, their children will be classified as descendants. |
| Immigrants of non-western** descent | Individuals born abroad, where neither parent is a Danish citizen or born in Denmark. If there is no information about any of the parents and the person was born abroad, the person is also perceived as an immigrant. |
| Descendants of non-western** immigrants | Individuals born in Denmark, where neither parent is both a Danish citizen and born in Denmark. If there is no information about any of the parents and the person is a foreign citizen, the person is also perceived as a descendant. When one or both parents born in Denmark obtain Danish citizenship, their children will not be classified as descendants, but as persons of Danish origin. However, if Danish-born parents both maintain a foreign citizenship, their children will be classified as descendants. |

***** Individuals with country of origin of Nordic countries, EU countries, Andorra, Liechtenstein, Monaco, San Marino, Switzerland, the United Kingdom, the Vatican City, Canada, the United States, Australia and New Zealand.

**Individuals with country of origin of all other countries than the countries defined by western heritage.

**Table S2. Overview of the International Classification of Diseases, 10th revision (ICD-10) codes included in the comorbidity covariate.**

| **Disease** | **ICD-10 codes** |
| --- | --- |
| Diabetes | E10-E14 |
| Adiposity | E65-E68 |
| Cancer | Z85 |
| Neurological diseases | G10-G14, G20-G23, G35-G37, G71-G73, G80-G83, G90-G91, G93-G96, G99, M51 (without G360, G902) |
| Kidney diseases | N180-N200Z, N18, Z992 |
| Hematological cancers | C81-C96, Z856-Z857 |
| Cardiovascular diseases | I20, I230-I259Z, I45-I499Z, I24, I50-I099Z, I340-I399Z, I05, I44, I21-I238Z, I000-I029Z, I30-I399Z, I26-I289Z, I40-I439Z, I50-I528Z, R01-R012B, I10-I59Z |
| Respiratory diseases | J400-J998Z, J40, J100-J229Z, J68, J430-J499 |
| Immune diseases | B200-B249Z, B20, Z21, D800-D899Z, Z923, Z926, Z941-Z949, Z94 (without Z945, Z947) |
| Other diseases | K700-K709, K70, E150-E909Z, D500-D649Z, D709-D779Z, D50, D650-D699Z, K710-K778Z, Q200-Q349Z, A150-A199Z, A15, Z902, Z905, Y90, Y900-Y919, Z251, |

Table S3. Coefficients and standard error by educational level and disposable income

|  | Coefficient^a^ | Std. error^a^ | Coefficient^b^ | Std. error^b^ | Coefficient^c^ | Std. error^c^ | Coefficient^d^ | Std. error^d^ |
| --- | --- | --- | --- | --- | --- | --- | --- | --- |
| Educational level |  |  |  |  |  |  |  |  |
| Master or Ph.D. | Reference | Reference | Reference | Reference | Reference | Reference | Reference | Reference |
| Bachelor | 0.25 | 0.0065 | 0.43 | 0.0066 | 0.39 | 0.0069 | 0.40 | 0.0070 |
| Secondary school | 0.83 | 0.0069 | 0.89 | 0.0073 | 0.65 | 0.0077 | 0.62 | 0.0078 |
| Vocational education | 0.31 | 0.0062 | 0.77 | 0.0064 | 0.82 | 0.0068 | 0.81 | 0.0069 |
| Primary school | 0.92 | 0.0060 | 1.34 | 0.0065 | 1.09 | 0.0069 | 1.05 | 0.0070 |
| Disposable income |  |  |  |  |  |  |  |  |
| >134,416 EUR | Reference | Reference | Reference | Reference | Reference | Reference | Reference | Reference |
| 94,091-134,416 EUR | 0.23 | 0.0317 | 0.15 | 0.0318 | 0.10 | 0.0320 | 0.09 | 0.0322 |
| 60,487-94,090 EUR | 0.80 | 0.0279 | 0.65 | 0.0279 | 0.49 | 0.0282 | 0.46 | 0.0283 |
| 33,605-60,486 EUR | 1.51 | 0.0274 | 1.30 | 0.0276 | 0.92 | 0.0279 | 0.87 | 0.0280 |
| <33,605 EUR | 1.84 | 0.0273 | 2.01 | 0.0930 | 1.43 | 0.0278 | 1.31 | 0.0280 |

a: Model 1: Unadjusted

b: Model 2: Adjusted for age and sex

c: Model 3: Adjusted for age, sex, immigration status, educational level and disposable income

d: Model 4: Adjusted for age, sex, immigration status, educational level, disposable income and history of SARS-CoV-2 infection
